# Validation of a Novel Mobile Application for Assessing Pediatric Tracheostomy Emergency Simulations

**DOI:** 10.1002/oto2.145

**Published:** 2024-07-04

**Authors:** Marc‐Mina Tawfik, Elliot Schiff, Roxanna Mosavian, Christine Campisi, Amanda Shen, Juan Lin, Alanna M. Windsor, Jacqueline Weingarten‐Arams, Sara H. Soshnick, Akira Nishisaki, Sangmo Je, Tensing Maa, Ilana Harwayne‐Gidansky, Regine M. Fortunov, Christina J. Yang, for the International Network for Simulation‐based Pediatric Innovation, Research, and Education (INSPIRE) Investigators

**Affiliations:** ^1^ Albert Einstein College of Medicine Bronx New York USA; ^2^ Department of Otorhinolaryngology–Head and Neck Surgery Montefiore Medical Center Bronx New York USA; ^3^ Department of Pediatrics, Division of Pediatric Critical Care Medicine Children's Hospital at Montefiore Bronx New York USA; ^4^ University of Pennsylvania Perelman School of Medicine Philadelphia Pennsylvania USA; ^5^ Department of Anesthesiology and Critical Care Medicine, Division of Pediatric Critical Care Medicine Children's Hospital of Philadelphia Philadelphia Pennsylvania USA; ^6^ Ohio State University College of Medicine Columbus Ohio USA; ^7^ Department of Pediatrics, Division of Pediatric Critical Care Medicine Nationwide Children's Hospital Columbus Ohio USA; ^8^ Department of Pediatrics, Division of Pediatric Critical Care Medicine, The Bernard & Millie Duker Children's Hospital Albany Medical Center Albany New York USA; ^9^ Albany Medical College Albany New York USA; ^10^ Department of Pediatrics, Division of Neonatology Baylor College of Medicine Houston Texas USA; ^11^ Texas Children's Hospital Houston Texas USA

**Keywords:** healthcare simulation, mobile applications, patient safety, quality improvement, tracheostomy, tracheotomy

## Abstract

**Objective:**

Pediatric tracheostomy is associated with high morbidity and mortality, yet clinician knowledge and quality of tracheostomy care may vary widely. In situ simulation is effective at detecting and mitigating related latent safety threats, but evaluation via retrospective video review has disadvantages (eg, delayed analysis, and potential data loss). We evaluated whether a novel mobile application is accurate and reliable for assessment of in situ tracheostomy emergency simulations.

**Methods:**

A novel mobile application was developed for assessment of tracheostomy emergency in situ simulation team performance. After 1.25 hours of training, 6 raters scored 10 tracheostomy emergency simulation videos for the occurrence and timing of 12 critical steps. To assess accuracy, rater scores were compared to a reference standard to determine agreement for occurrence or absence of critical steps and a timestamp within ±5 seconds. Interrater reliability was determined through Cohen's and Fleiss' kappa and intraclass correlation coefficient.

**Results:**

Raters had 86.0% agreement with the reference standard when considering step occurrence and timing, and 92.8% agreement when considering only occurrence. The average timestamp difference from the reference standard was 1.3 ± 18.5 seconds. Overall interrater reliability was almost perfect for both step occurrence (Fleiss' kappa of 0.81) and timing of step (intraclass correlation coefficient of 0.99).

**Discussion:**

Using our novel mobile application, raters with minimal training accurately and reliably assessed videos of tracheostomy emergency simulations and identified areas for future refinement.

**Implications for Practice:**

With refinements, this innovative mobile application is an effective tool for real‐time data capture of time‐critical steps in in situ tracheostomy emergency simulations.

Pediatric tracheostomy is associated with high morbidity and mortality, with complication rates ranging 12.6% to 30%.^1^, ^2^, ^3^, ^4^ Additionally, tracheostomy complication‐associated mortality rates are tenfold higher among pediatric patients compared to adults.^5^, ^6^ Tracheostomy‐related complications (eg, accidental decannulation and obstruction) can lead to loss of airway and subsequent hypoxic brain injury if not managed quickly and correctly.^5^, ^6^, ^7^, ^8^, ^9^ However, knowledge and understanding of tracheostomy care varies widely, even among healthcare providers who routinely care for patients with tracheostomies.^10^, ^11^, ^12^, ^13^ In situ simulation, the use of simulated scenarios in the real clinical environment, is a training tool to improve multidisciplinary team dynamics and management of critical events. It can also measure system‐level performance and identify latent safety threats (LSTs), and organizational or environmental issues that make a system more prone to human error.^14^, ^15^, ^16^, ^17^, ^18^, ^19^, ^20^, ^21^, ^22^, ^23^

Assessment of in situ simulation performance, including objective time‐based evaluation of provider performance and identification of LSTs, can be accomplished by a variety of methods including checklists, structured debriefing, and video review.^24^, ^25^ While video review can be beneficial as it allows multiple assessments of the simulation, it is not without disadvantages.^26^, ^27^ Storage of video files introduces risk of file corruption and loss, and a secure storage method must be ensured before a study can begin. As video review is inherently retrospective, time must also be allotted for review, which may be inconvenient for reviewers and lead to delays in analysis.^28^, ^29^ Additionally, video review does not capture events that occurred outside the camera view and may induce anxiety and consequently influence participants' behaviors or willingness to participate during simulations.^30^ Thus, the ability to accurately assess in situ simulations in real‐time may offer advantages in both quality and convenience of data collection. Finally, a means to standardize reviews is desired to allow for better comparison of simulation performance over time and across diverse settings.

While mobile applications have been successfully used during in situ simulations for resuscitation scenarios, their use in tracheostomy emergency scenarios remains understudied.^31^, ^32^, ^33^, ^34^, ^35^ NeoCHART+™ is a mobile‐based software suite with modular components to support clinical care, education, and research. The NeoCHART+™ for PEAK‐II application was developed to support the Children's Hospital of Philadelphia's multi‐institutional PEAK‐II (Prevention of Errors in Acute Conditions in Kids) initiative to study and identify systems‐based LSTs while caring for children with acute, life‐threatening conditions. NeoCHART+^TM^ for PEAK‐II uses a custom version of the research module to capture key team actions with an exact timestamp and allows the collection of data from multiple sites into REDCap.

In 2023, the PEAK‐II initiative was expanded to target tracheostomy emergencies (“PEAK‐II Trach”). Within PEAK‐II Trach, the NeoCHART+^TM^ for PEAK‐II application was adapted for assessment of tracheostomy emergencies, in addition to the respiratory compromise scenario for which it was first piloted.^31^ This expansion allows users to assess performance of participants during in situ simulations of tracheostomy emergencies by selecting if key team actions in a tracheostomy emergency were performed and the time these actions were performed. Actions included in the application are based on international consensus recommendations regarding the most critical components (“steps”), of an emergent tracheostomy change.^36^ This study aims to establish accuracy and reliability of the NeoCHART+^TM^ for PEAK‐II mobile application for evaluating team performance of critical steps during simulated pediatric tracheostomy emergencies.

## Methods

### Overview

To assess the accuracy and reliability of the NeoCHART+™ for PEAK‐II application for evaluating simulated pediatric tracheostomy emergencies (PEAK‐II Trach), 6 raters evaluated 10 video‐recorded simulations, using the application to determine if and when 12 key tracheostomy change steps occurred. Accuracy was determined by comparing rater responses to reference values independently established by 2 other study members, and reliability was assessed through measures of interrater reliability.

### Simulation Video Sourcing

This study was approved by the Albert Einstein College of Medicine Institutional Review Board (IRB) (protocols 2018‐9421 and 2022‐14457). Written consent was obtained from all simulation participants. The simulations were video‐recorded at the Children's Hospital at Montefiore from November 2018 to March 2023 as part of ongoing quality improvement and educational efforts in the pediatric intensive care unit (PICU), neonatal intensive care unit (NICU), pediatric emergency department (ED), pediatric post‐anesthesia care unit (PACU), and pediatric floors. Two authors (RM and CC) selected 10 videos with acceptable audio and visual quality (eg, no obstructed views, complete video), avoiding duplicated participants, and representing the span of clinical settings and years of simulation, as described in Table 1.

**Table 1:** Pediatric Tracheostomy Emergency Simulation Video Description and Reference Values

	Video	1	2	3	4	5	6	7	8	9	10
	Location	PICU	NICU	ED	ED	PICU	Floor	Floor	PACU	NICU	NICU
	Year	2021	2018	2022	2018	2020	2023	2023	2023	2023	2023
Time of step^a^ (seconds)	Step 1 “Assesses Breathing”	23	34	95	41	8		22	119	55	
	Step 2 “Calls for Help”	16		165	205			166	93	87	98
	Step 3 “Suctions”		90	51	147	47	28		18	47	94
	Step 4 “Trach Problem”	61	74	232	131	122	91		12	64	222
	Step 5 “Trach Size‐Length‐Type”			69		135					
	Step 6 “Maskable”			203							
	Step 7 “Shoulder Roll”									120	
	Step 8 “Removes Trach”	104	261			190	137	136	197	235	
	Step 9 “Bag Mask”	99		214						196	
	Step 10 “Inserts Trach”	166	266			197			204	239	
	Step 11 “Trach Vent”	170	272			203	151		212	248	
	Step 12 “Trach Ties”						251			284	

Abbreviations: ED, pediatric emergency department; Floor, pediatric medicine floor; NICU, neonatal intensive care unit; PACU, pediatric post‐anesthesia care unit; PICU, pediatric intensive care unit.

^a^ Occurrence and timing of steps determined through consensus video review by 2 authors as reference standard.

### Simulation Scenario Overview

Each simulation scenario included a multidisciplinary simulation team of each unit's typical first responders to a tracheostomy emergency, such as resident and/or fellow physicians, physician assistants, nurse practitioners, nurses, and respiratory therapists. The scenario comprised an infant with a chronic tracheostomy admitted for an upper respiratory infection and experiencing sudden oxygen desaturation due to a tracheostomy tube which was partially dislodged and obstructed with a mucus plug. Once started, simulated oxygen saturation dropped by 10% every 60 seconds until ventilation was restored. The scenario ended once the tracheostomy tube had been replaced and secured with tracheostomy ties and ventilation re‐established, or if 5 minutes had elapsed from the scenario start.

We utilized a Life/form Special Needs Infant manikin (Nasco Healthcare) with the existing tracheostomy enlarged with a 7/32 drill bit to fit a size 3.5 pediatric tracheostomy tube. For the simulations, the tracheostomy tube was occluded and partially dislodged (lumen blocked with putty, with several drain sponges underneath the flanges and loose tracheostomy ties). Participants were instructed to use the equipment and resources normally available in their clinical setting, and the simulation environment was set up in accordance with unit standards. For simulations conducted in an ICU, the scenario also included a video of a ventilator alarm reflecting low volumes and a high peak pressure played from the simulation start.^17^

### Establishing Critical Steps and Data Definitions

Critical action steps to perform during the in situ simulations were selected by the study team through discussion and review of published international consensus recommendations for pediatric tracheostomy emergency readiness.^36^, ^37^, ^38^ Action steps were selected with consideration to both their importance and feasibility of observation during simulation. Data definitions were developed to standardize assessment. A pilot review by MT and ES of 5 simulation videos with the NeoCHART+^TM^ for PEAK‐II application further refined the selection of steps and their data definitions to mitigate ambiguity and eliminate steps too closely timed to be assessed. The final 12 critical action steps and corresponding data definitions are summarized in Table 2. Notably, for each critical step, Table 2 includes the full name of the step, the name as shown in the NeoCHART+™ for PEAK‐II application, and an abbreviated short‐hand name. For ease of reference, the abbreviated short‐hand names will be used throughout this manuscript unless otherwise noted.

**Table 2:** Critical Steps and Data Definitions Included in the NeoCHART+™ Application

Step	Step full name (*name in application*)^a^	Shorthand step name^b^	Step definition
1	Assesses Breathing (auscultation/chest rise) *Assesses Breathing*	“Assesses Breathing”	Auscultation or asks facilitator about chest rise
2	Calls for Help *Calls for Help*	“Calls for Help”	At least one additional person/team verbally called/paged to assist with potential tracheostomy tube change/emergency OR An appropriate response team paged for assistance (i.e., ENT, rapid response/code team, critical care)
3	Suctions Tracheostomy *Suctions Trach*	“Suctions”	Suction catheter inserted into tracheostomy to correct targeted suction depth (approximately the length of the tracheostomy tube shaft)
4	States Problem is Related to the Tracheostomy Tube *Says is a Trach Problem*	“Trach Problem”	Makes a reference to the tracheostomy tube being plugged, dislodged, or otherwise malfunctioning *Examples: “I think the trach might be plugged,” “I think the trach might be out/dislodged,” or “I think there is something wrong with the trach tube”*
5	Says Tracheostomy Size‐Length‐Type *Says Trach Size‐Length‐Type*	“Trach Size‐Length‐Type”	For credit, must state all 3 descriptors *Example: “3.5 cuffless pediatric Shiley”*
6	Asks if Maskable *Asks if Maskable*	“Maskable”	Verbal statement asking whether patient is maskable or intubatable or “not a critical airway”
7	Places Shoulder Roll *Places Shoulder Roll*	“Shoulder Roll”	Shoulder roll placed and manikin laid back down
8	Removes Old Tracheostomy Tube *Removes Old Trach*	“Removes Trach”	Tube removed from manikin
9	Starts Facemask Bag Ventilation *Starts Facemask Bag Ventilation*	“Bag Mask”	Bag is squeezed while held to the face *Only first time recorded for analysis*
10	Inserts Identical New Tracheostomy Tube (with obturator) *Inserts Identical Trach*	“Inserts Trach”	New tube is fully inserted into the manikin stoma in a downward curved motion, until tube flanges are flush with the skin (mark once tube phalanges become flush) *Do not give credit if a tube of a different size, length, and/or type is inserted*
11	Starts New Tracheostomy Tube Ventilation (bag or vent) *Starts New Trach Ventilation*	“Trach Vent”	1st time bag is squeezed while attached to the new tracheostomy tube OR ventilator attached to newtube *Do not give credit if ventilating tube that is still obstructed*
12	Ensures Tracheostomy Ties Tight *Ensures Trach Ties Tight*	“Trach Ties”	Both sides of the tie are completely secure around the manikin's neck (such that one finger could be inserted under the ties at the neck)

^a^ Step name as shown in the NeoCHART+™ application.

^b^ Abbreviated step name for ease of reference.

### Establishing Reference Standard Values

Two authors (RM and CC) independently reviewed 10 videos to establish reference standard values for the 12 critical steps. These authors were not among the 6 raters for the primary analysis. Videos could be paused and replayed freely. Each step was assessed for occurrence or absence of successful completion and the time from the start of the scenario that the step completion occurred.

Resolution of lack of consensus for step occurrence, or a timestamp difference of greater than 1 second, was adjudicated by joint review of the videos and data definitions until consensus was reached. Across the 10 videos, 22/120 steps (18.33%) required adjudication by joint review by RM and CC to establish consensus.

### NeoCHART+™ for PEAK‐II Application

**Figure 1:**
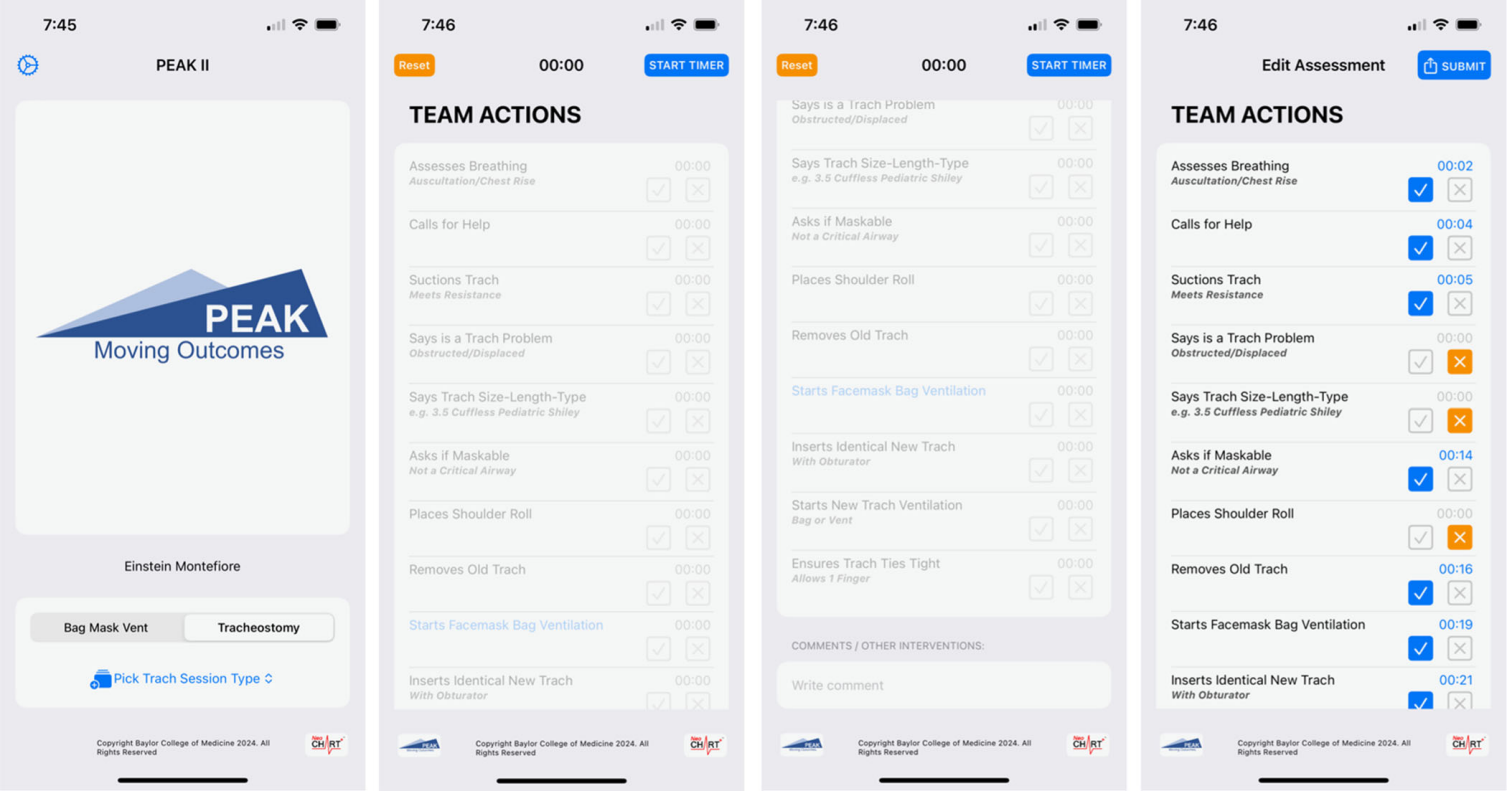
Screenshots of NeoCHART+™ for PEAK‐II Interface. From left to right: welcome page to select scenario and review type, list of critical steps with start timer button, final review page prior to REDCap submission.

NeoCHART+™ for PEAK‐II, hereafter referred to as NeoCHART+™, is an iOS application developed by author RF. Users were instructed to start the timer at the simulation start and check‐off critical steps as they were completed. Any steps marked by the user as conducted were automatically assigned a timestamp. After ending the scenario, users were able to review their data before submitting to the REDCap database. Additionally, users submitted comments describing participant performance and the rationale for the score given. Screenshots of the NeoCHART+^TM^ interface are shown in Figure 1.

### Rater Selection, Training, and Video Review with NeoCHART+™

Six raters with varied training backgrounds were chosen and included 2 pediatric otolaryngologists (AW and CY), 2 pediatric intensivists (JW and SS), and 2 medical students (MT and ES) with extensive involvement in tracheostomy quality improvement research. All raters had at least 1year of training and experience conducting pediatric tracheostomy simulations. All raters met for 1 hour to review the data definitions and received 15‐min of video‐recorded training material demonstrating examples of “excellent,” “good,” and “poor” performance simulations to practice scoring using NeoCHART^TM^. Training materials were produced at the Montefiore Einstein Center for Innovation in Simulation, with a description of the 3 videos in Supplemental Table S1, available online. Following training, each rater assessed the 10 study videos with NeoCHART+^TM^. Each video was watched once without pausing or rewinding. Data were censored at 5 minutes (the end of the scenario).

### Data Analysis

Rater accuracy was assessed by comparing rater responses to the reference standard for the occurrence and timing of each step. Time‐based agreement was defined as agreement with the reference standard within ±5 seconds. A time difference of 5 seconds was chosen a priori to account for the time needed to interact with the application and with the assumption that this time difference would be clinically negligible.

To assess which factors most impacted accuracy, a generalized linear mixed‐effects regression (GLMER) model was fitted using R package “lme4” to examine the effect of video, step, and rater experience level on probability of time‐based agreement.^39^ Using the model, the predicted probability of time‐based agreement of each factor (video, step, and rater experience level), marginalized over the different levels of other factors, was estimated. The odds ratio and its 95% confidence interval of a time‐based agreement for each video, step, and rater experience level, compared to respective reference value, were also determined. Statistical significance was set at *P* < .05. The highest time‐based agreement by experience level and video were chosen as reference values in the multivariable regression analysis. The step with both the highest time‐based agreement and a high frequency of occurrence (70% of videos) was chosen as the reference value.

Interrater reliability was determined by comparing their responses for both step occurrence and timing. Cohen's kappa (between pairs of raters of the same experience level) and Fleiss' kappa (between all raters) were calculated for the assessment of step occurrence. Intraclass correlation coefficient (ICC) was calculated for assessments of time within experience levels and among all raters for all instances in which a timestamp was assigned by all raters being compared. ICC values and their 95% confidence intervals were calculated based on a mean‐rating, absolute‐agreement, 2‐way mixed‐effects model.

To better understand the impact of a step that was found by GLMER to have poor predicted probability of time‐based agreement, a post hoc sensitivity analysis was performed in which that step was excluded. Accuracy and reliability results are therefore presented below with and without that step for comparison.

GLMER was performed in R, version 4.0.3. Cohen's kappa, Fleiss' kappa, and ICC determinations were performed in SPSS, version 28.0.1.0. All other calculations were performed in Microsoft Excel, version 16.78.

## Results

### Video Characteristics and Reference Standard Review

Simulation video data and reference standard values are displayed in Table 1. The most frequently performed steps were steps 4, “Trach Problem,” (90% of videos), 1, “Assesses Breathing,” (80% of videos), and 3, “Suctions,” (80% of videos). Steps 6, “Maskable,” and 7, “Shoulder Roll,” were each correctly performed in only 1 video (10%).

### Accuracy—Initial Analysis

**Figure 2:**
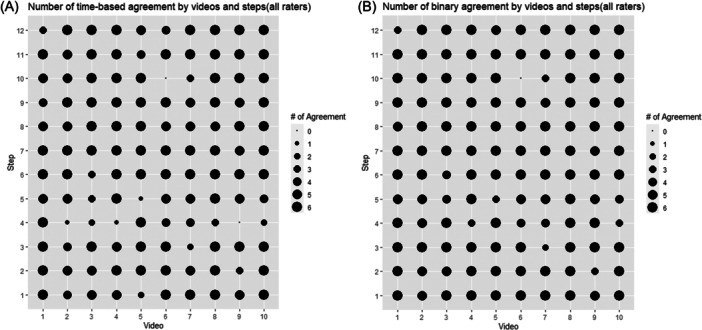
(A) Number of time‐based agreement and (B) step occurrence agreement by video and step for all raters (n = 6).

When assessing time‐based agreement across all videos, steps, and raters, 86.0% of values recorded with NeoCHART+^TM^ agreed with the reference standard (Table 3 and Figure 2A). Agreement was highest for otolaryngologists (90.0%), followed by medical students (86.7%) and then pediatric intensivists (81.3%) (Table 3).

**Table 3:** Initial Measures of NeoCHART+™ Accuracy (All Videos and Steps)

Rater	Time‐based agreement, %	Step occurrence agreement, %	Average difference from reference standard, Sec ± SD (number of timestamp comparisons)
ENT #1	90.8	96.7	+1.3 ± 9.6 (56)
ENT #2	89.2	96.7	+2.9 ± 23.4 (57)
All ENT	90.0	96.7	+2.1 ± 17.9 (113)
CC #1	76.7	89.2	+3.1 ± 17.0 (55)
CC #2	85.8	90.8	+2.1 ± 22.8 (53)
All CC	81.3	90.0	+2.6 ± 20.0 (108)
MS #1	85.0	91.7	−4.1 ± 19.3 (54)
MS #2	88.3	91.7	+2.4 ± 15.5 (55)
All MS	86.7	91.7	−0.8 ± 17.7 (109)
**All raters**	**86.0**	**92.8**	**1.3** ± **18.5 (330)**

Abbreviations: CC, pediatric critical care attending physician; ENT, pediatric otolaryngology attending physician; MS, 3rd or 4th year medical student.

Step occurrence agreement was 92.8% (Table 3 and Figure 2B) with reference standard. Otolaryngologists had the greatest agreement at 96.7%, followed by 91.7% and 90.0% for students and intensivists, respectively.

**Figure 3:**
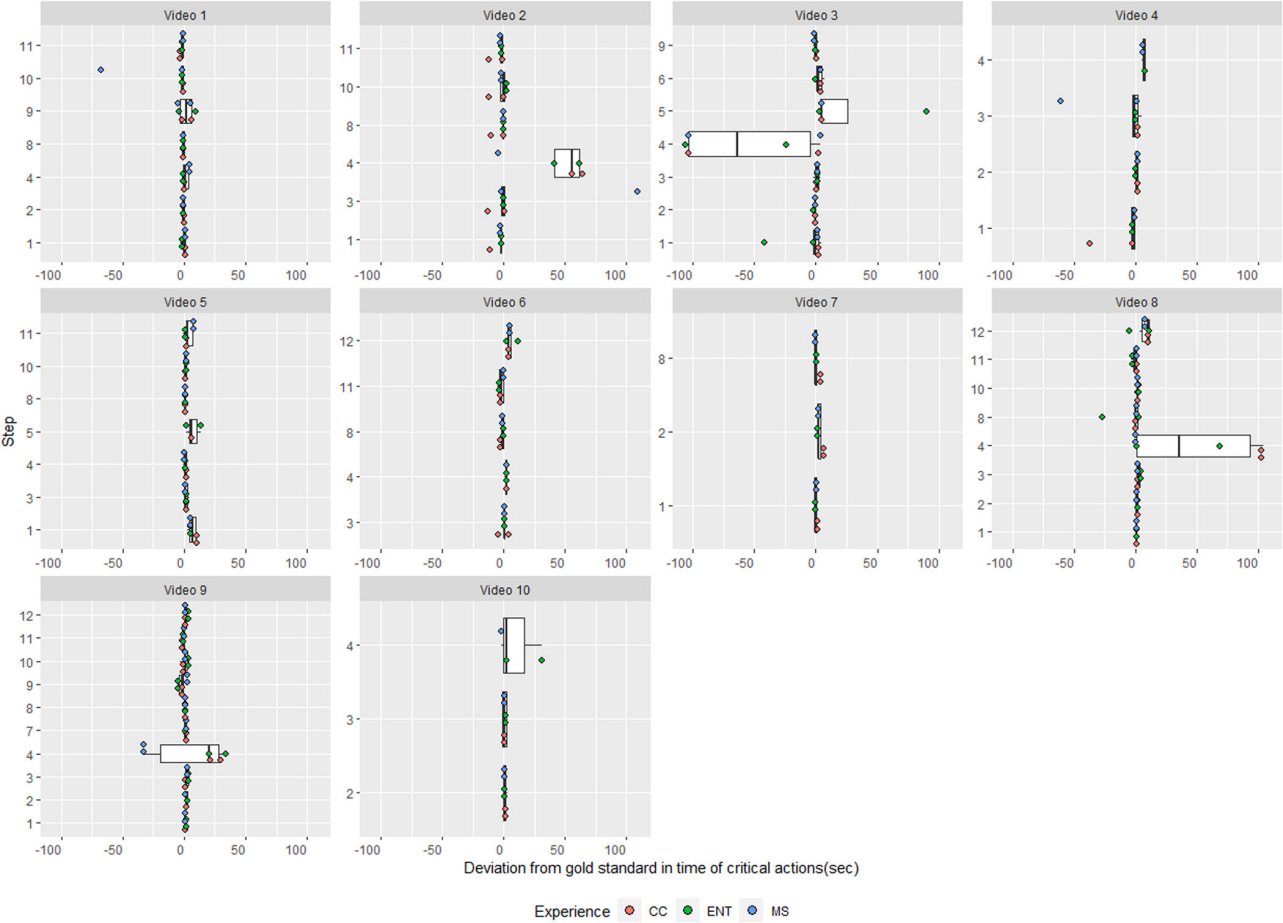
Timestamp deviation from reference standard. Negative and positive numbers are steps recorded earlier or later than the reference standard, respectively. CC, pediatric critical care attending; ENT, pediatric otolaryngology attending; MS, 3rd or 4th year medical student.

When both the reference standard and a NeoCHART+^TM^ rater assigned a timestamp for the same video and step, raters differed from the reference standard by +1.3 ± 18.5 seconds (n = 330) (Table 3 and Figure 3). Otolaryngologists differed from the reference standard by +2.1 ± 17.9 seconds (n = 113), intensivists by +2.6 ± 20.0 seconds (n = 108), and students by −0.8 ± 17.7 seconds (n = 109).

Measures of accuracy by video and step are summarized in Supplemental Table S2, available online.

### Accuracy—Multivariate Regression Analysis

In multivariate regression analysis for time‐based agreement, video selection did not significantly affect agreement (*P* = .41) (Table 4). However, statistically significant differences were found due to rater experience levels and steps (*P* = .004 and *P* < .001, respectively). Compared to otolaryngologists, intensivists were found to have a small but statistically significant difference in probability of time‐based agreement; odds ratio [OR] = 0.32 (0.16‐0.63), *P* = .001. Compared to step 8, “Removes Trach,” step 4, “Trach Problem,” was found to have a significant difference in probability of time‐based agreement; OR = 0.04 (0.01‐0.26), *P* < .001.

**Table 4:** Generalized Linear Mixed‐Effects Regression Results for Time‐Based Agreement

Variable	Predicted agreement, % (95% CI)	OR of predicated agreement (95% CI)	*P* value
Video^a^			.41
1	96 (84‐9)	0.21 (0.02‐1.99)	.17
2	90 (72‐97)	0.09 (0.01‐0.81)	.03
3	93 (79‐98)	0.14 (0.01‐1.30)	.08
4	98 (90‐100)	0.44 (0.04‐4.87)	.50
5	94 (79‐98)	0.14 (0.02‐1.35)	.09
6	94 (80‐98)	0.15 (0.02‐1.42)	.10
7	92 (76‐98)	0.11 (0.01‐0.98)	.05
8	99 (94‐100)	*Ref*	*Ref*
9	91 (74‐97)	0.10 (0.01‐0.99)	.05
10	99 (92‐100)	0.68 (0.06‐8.20)	.76
Rater experience^b^			.004
ENT	97 (94‐99)	*Ref*	*Ref*
CC	92 (86‐96)	0.32 (0.16‐0.63)	.001
MS	96 (92‐98)	0.61 (0.30‐1.22)	0.16
Step^c^			<.001
1	93 (76‐98)	0.56 (0.08‐4.19)	.58
2	96 (84‐99)	1.08 (0.13‐8.88)	.94
3	96 (82‐99)	0.96 (0.12‐7.82)	.97
4	48 (22‐74)	0.04 (0.01‐0.26)	<.001
5	83 (59‐94)	0.21 (0.03‐1.42)	.11
6	99 (91‐100)	3.53 (0.30‐41.29)	.32
7	100 (94‐100)	9.81 (0.52‐186.47)	.13
8	96 (84‐99)	*Ref*	*Ref*
9	98 (88‐100)	1.71 (0.19‐15.32)	.63
10	92 (73‐98)	0.49 (0.07‐3.66)	.49
11	98 (91‐100)	2.77 (0.27‐28.71)	.39
12	96 (84‐99)	1.07 (0.13‐8.74)	.95

Abbreviations: CC, pediatric critical care attending physician; ENT, pediatric otolaryngology attending physician; MS, 3rd or 4th year medical student.

^a^ All steps and raters.

^b^ All videos and steps.

^c^ All videos and raters.

### Accuracy—Sensitivity Analysis

Given the significant difference in time‐based agreement between step 4, “Trach Problem,” and other steps, a post hoc sensitivity analysis was performed to assess application accuracy with step 4 excluded. This improved overall time‐based agreement to 89.4% (Table 5). Agreement improved to 93.6%, 89.1%, and 85.5% among otolaryngologists, students, and intensivists, respectively. Multivariate regression analysis with step 4 excluded found no statistically significant difference in probability of agreement based on videos or steps (Supplemental Table S3, available online). A statistically significant difference in probability of agreement remained between intensivists and otolaryngologists; OR = 0.29 (0.13‐0.63), *P* = .002.

**Table 5:** Sensitivity Analysis of NeoCHART+™ Accuracy (All Videos and Steps 1‐3, 5‐12)

Rater	Time‐based agreement, %	Step occurrence agreement, %	Average difference from reference standard, Sec ± SD (number of timestamp comparisons)
ENT #1	93.6	96.4	+0.5 ± 7.2 (47)
ENT #2	93.6	97.3	+1.5 ± 13.7 (49)
All ENT	93.6	96.8	+1.0 ± 11.0 (96)
CC #1	80.0	90.9	−0.5 ± 5.0 (49)
CC #2	90.9	92.7	+0.6 ± 6.1 (46)
All CC	85.5	91.8	+0.01 ± 5.5 (95)
MS #1	87.3	91.8	−2.0 ± 13.8 (45)
MS #2	90.9	93.6	+3.0 ± 15.6 (49)
All MS	89.1	92.7	+0.6 ± 14.9 (94)
**All raters**	**89.4**	**93.8**	+0.6 ± **11.1 (285)**

Abbreviations: CC, pediatric critical care attending physician; ENT, pediatric otolaryngology attending physician; MS, 3rd or 4th year medical student.

Removing step 4 from the analysis improved overall step occurrence agreement with the reference standard to 93.8%, with an average timestamp difference from the reference standard of +0.6 ± 11.1 seconds (n = 285), as summarized in Table 5.

### Reliability—Initial Analysis

Cohen's kappa for step occurrence agreement was 0.90, 0.73, and 0.83 for otolaryngologists, intensivists, and medical students respectively (Table 6). This suggests almost perfect reliability for otolaryngologist raters, substantial for intensivist raters, and almost perfect for medical student raters.^40^ Almost perfect reliability was found for step occurrence agreement among all raters with a Fleiss kappa of 0.81. For timestamps, reliability was almost perfect with ICCs of 0.99 for otolaryngologists and intensivists, 0.98 for medical students, and 0.99 among all raters.^41^

**Table 6:** Initial Measures of NeoCHART+™ Reliability (All Videos and Steps)

Rater type	Step occurrence agreement Cohen's Kappa	Step occurrence agreement Fleiss' Kappa (95% CI)	Timestamp Intraclass correlation coefficient (95% CI; number of comparisons)
ENT	0.90		0.99 (0.98‐0.99; 55)
CC	0.73		0.99 (0.98‐0.99; 53)
MS	0.83		0.98 (0.95‐0.99; 55)
**All raters**		**0.81 (0.76‐0.86)**	**0.99 (0.99‐1.0; 47)**

Abbreviations: CC, pediatric critical care attending physician; ENT, pediatric otolaryngology attending physician; MS, 3rd or 4th year medical student.

### Reliability—Sensitivity Analysis

Removal of step 4, “Trach Problem,” from the analysis resulted in Cohen's kappa for step occurrence agreement of 0.91, 0.75, and 0.89 for otolaryngologists, intensivists, and students, respectively (Table 7). Fleiss' kappa improved to 0.84. ICCs of timestamps were 1.0 for otolaryngologists and intensivists, 0.98 for students, and 1.0 among all raters.

**Table 7:** Sensitivity Analysis of NeoCHART+™ Reliability (All Videos and Steps 1‐3, 5‐12)

Rater type	Step occurrence agreement Cohen's Kappa	Step occurrence agreement Fleiss' Kappa (95% CI)	Time stamp Intraclass correlation coefficient (95% CI; number of comparisons)
ENT	0.91		1.0 (1.0‐1.0; 47)
CC	0.75		1.0 (1.0‐1.0; 47)
MS	0.89		0.98 (0.97‐0.99; 49)
**All raters**		**0.84 (0.79‐0.89)**	**1.0 (1.0‐1.0; 42)**

Abbreviations: CC, pediatric critical care attending physician; ENT, pediatric otolaryngology attending physician; MS, 3rd or 4th year medical student.

## Discussion

While mobile applications have been previously developed to teach tracheostomy care skills^42^ and record emergency airway procedures,^43^ to our knowledge this is the first utilization of a mobile application to evaluate performance during simulated tracheostomy emergencies. Our work highlights the potential utility of real‐time data collection with software as an alternative to post‐simulation video review and offers some areas for future refinement.

Step occurrence agreement of 92.8% with the reference standard suggests that raters using NeoCHART+^TM^ are capable of accurately recording the occurrence of key steps as they are completed without the need for pausing and rewinding the video. In the largest example of poor agreement (0% agreement for video 6, step 10, “Inserts Trach”), participants used a down‐size tracheostomy tube, the tube they found first. All raters gave credit, but the reference standard did not. This suggests ambiguity in the best clinical practice, which potentially could be accommodated by the reference standard, as well as potential difficulty determining if the correct tube is being used in a simulation unless explicitly verbalized. The decision to use a strict definition for step 10, “Inserts Trach”, was initially made to standardize scoring and increase comparability. However, since in a tracheostomy emergency it can be appropriate to use the first available tube, we could consider adjusting the data definition for step 10 to reflect this accepted clinical practice.

The average timestamp difference of +1.3 seconds suggests that raters are largely able to accurately assess the timing of key steps, however, the large standard deviation of ±18.5 seconds and Figure 3 indicate outliers. Forty‐nine of 330 (14.48%) values that could be compared exceeded the ±5 seconds threshold from the reference value, and 20 of 49 (40.82%) occurred in step 4, “Trach Problem.” We hypothesize that step 4 is uniquely challenging given its subjectivity and the need for participants to verbalize a cognitive process. The limitations of measuring cognitive steps like step 4 may be potentially overcome by assessing only occurrence and not timing, scoring separately, explicitly asking participants to verbalize thoughts as part of simulation instructions, and/or clarification of criteria to determine appropriate vocalization. For video 5, step 5, “Trach Size‐Length‐Type,” (16.7% time‐based agreement among raters), participants did not confidently state if the tube was cuffed or uncuffed; this led some raters not to award credit, and others to award credit late. Again, explicit instruction to verbalize items not visible by video (eg, tracheostomy size or cuff) may overcome this limitation, but real‐time assessment with in‐person visualization may also reduce this issue.

The positive direction for the timestamp difference is expected given the time required for the rater to interact with the application interface after witnessing step completion. Interquartile ranges of (25th percentile: −1.0 seconds, 75th percentile: +2.0 seconds) both with and without step 4 indicate that most time errors are positive but a meaningful subset of steps are marked early. This small, negative timestamp difference likely represents raters marking steps at their start rather than their completion, which will require more explicit rater training to address. In video 2, 1 rater, with 41.7% time‐based agreement for that video, had 5 of 6 timestamps with a consistent difference of 10 to 13 seconds, likely representing an error in pressing the start timer button at the beginning of the simulation.

The discrepancy in accuracy and interrater reliability by rater experience may have been driven by differing levels of experience with both tracheostomy care and the NeoCHART+^TM^ applications. Relative to other raters, otolaryngologists have the greatest exposure and experience with tracheostomy care, allowing them to assess simulation performance more accurately. While intensivists have greater experience than medical students, the medical students in this study had significantly greater familiarity with the NeoCHART+^TM^ application given participation in setting the data definitions and a pilot analysis with the application. Student performance may also have been aided by strong interests in otolaryngology, familiarity with tracheostomy care, and experience facilitating similar simulations. This comfort with the application and data definitions likely allowed the students to approach the level of agreement found for otolaryngologists. This suggests that relative novices can achieve similar results to subject experts with increased exposure to the NeoCHART+^TM^ application, simulation, and subject matter, as well as the need for explicit and specific guidance during rater training.

Other studies of mobile applications during healthcare simulations have largely studied their use as implementation/quality improvement tools rather than their validity as assessment/scoring tools.^44^, ^45^, ^46^, ^47^, ^48^, ^49^ In the absence of a comparator, we hypothesize that in most instances the benefits of real‐time assessment with NeoCHART+^TM^ outweigh increased accuracy with manual review. Specifically, NeoCHART+^TM^ may minimize the need for video recording and processing, which can be resource‐intensive, be prone to issues of poor quality, data loss, and prolonged data storage, can require informed consent, may increase anxiety among simulation participants, and may serve as a barrier to participation among individuals who would prefer not to be recorded. Furthermore, almost perfect interrater reliability among raters (Fleiss kappa of 0.81 for step occurrence and 0.99 for step timing) suggests that NeoCHART+^TM^ can be a source of standardized evaluations of simulation performance that can be easily compared.

A limitation of this study is the need to use NeoCHART+™ with video review to compare to traditional methods and to allow all raters to assess the same simulations. Review was therefore subject to many of the challenges that NeoCHART+™ seeks to overcome, such as events outside of view of the camera. Additionally, there may be differences in how NeoCHART+™ performs when used in‐person during a simulation versus post hoc video review. This study was also not able to assess how NeoCHART+™ may perform if a rater was balancing other roles (eg, giving prompts) during the simulation. As each video was only reviewed once per rater, intra‐rater reliability could not be determined. Some steps, such as step 6, “Maskable,” and 7, “Shoulder Roll,” were rarely performed by teams in the videos reviewed, which therefore limited full assessment and validation of those steps. Additionally, the application is only available for iOS at this time, which could limit use in some settings. Lastly, our study included simulations from a single institution and therefore may not account for inter‐institutional nuances in simulation facilitation. We aim to overcome these limitations by continuing to study NeoCHART + ™ as it is deployed in our group's ongoing multi‐institutional PEAK‐II Trach study.

## Implications for Practice

### Clinical Implications

This study has implications for both PEAK‐II Trach (and within that, the continued development of NeoCHART+^TM^ for PEAK‐II) and for others developing applications for healthcare simulations.

Our findings suggest that NeoCHART+™ is accurate and reliable for evaluating performance in pediatric tracheostomy emergency simulations while identifying areas for additional refinement. Furthermore, similar performance between otolaryngologists and the medical students in our study suggests that increased familiarity with the application can bridge limitations in clinical experience, broadening applicability. Relatedly, we also hypothesize that more explicit rater training can improve rater adherence to data definitions across experience levels. The use of NeoCHART+™ during simulations may therefore reduce the need for video review. This may decrease requisite time and resources, allow more rapid access to results, and remove barriers to simulation participation.

Improved accuracy and interrater reliability in sensitivity analyses excluding step 4, “Trach Problem,” suggest that assessments of team performance should minimize subjectivity in data definitions. When assessing mental modeling, occurrence may be more meaningful than timing. Furthermore, data definitions for steps that include multiple attributes (eg, step 5, “Trach Size‐Length‐Type”) and the presence or absence of partial credit should be explicit parts of rater training to ensure consistency. Clear scenario start cues and an emphasis on marking steps upon their completion (and not their start) are essential to avoid systematic errors related to timing during rating.

### Future Directions

Future directions include the refinement, deployment, and continued study of the NeoCHART+™ application for simulated pediatric tracheostomy emergencies, as well as inclusion of other scenarios (eg, new tracheostomy tube unable to be placed, necessitating endotracheal intubation) and adaptation for use in simulated adult tracheostomy emergencies.

Refinement and development of accessible and clear operational definitions for steps, as well as more explicit rater training, will likely further improve the accuracy and reliability of the application. Refinements will target steps with weaker levels of agreement with the reference standard and amongst raters.

Following refinement, NeoCHART+^TM^ for PEAK‐II will be deployed at multiple institutions as part of the PEAK‐II Trach initiative to identify and address LSTs affecting the care of pediatric tracheostomy patients. Raters will be trained on a standardized set of videos prior to using NeoCHART+^TM^ in the field; based on overall performance in this study, a benchmark of 85% accuracy on 3 training videos will establish “trained rater” status.

## Author Contributions

**Marc‐Mina Tawfik**, design, conduct, analysis, composition and approval of final manuscript; **Elliot Schiff**, design, conduct, analysis, composition and approval of final manuscript; **Roxanna Mosavian**, design, conduct, analysis, composition and approval of final manuscript; **Christine Campisi**, design, conduct, composition and approval of final manuscript; **Amanda Shen**, design, conduct, analysis, composition and approval of final manuscript; **Juan Lin**, analysis, approval of final manuscript; **Alanna M. Windsor**, conduct, approval of final manuscript; **Jacqueline Weingarten‐Arams**, conduct, approval of final manuscript; **Sara H. Soshnick**, conduct, approval of final manuscript; **Sangmo Je**, conceptualization, design, approval of final manuscript; **Akira Nishisaki**, conceptualization, design, approval of final manuscript, senior supervision; **Tensing Maa**, conceptualization, design, approval of final manuscript; **Ilana Harwayne‐Gidansky**, conceptualization, design, approval of final manuscript; **Regine M. Fortunov**, NeoCHART+^TM^ application development, conceptualization, design, composition and approval of final manuscript, senior supervision; **Christina J. Yang**, conceptualization, design, conduct, composition and approval of final manuscript, senior supervision.

## Disclosures

### Competing interests

None.

### Funding sources

Dr. Yang was a Clinical Research Training Program scholar supported by the National Institutes of Health (NIH)/National Center for Advancing Translational Science (NCATS) Einstein Montefiore CTSA (Clinical and Translational Science Awards) Grant Number UL1TR001073, July 2021–May 2023. The research described was also supported by the Children's Hospital at Montefiore Mid‐senior Career Research Award.

## Supporting information

Supplementary information.

Supplementary information.

Supplementary information.

Supplementary information.
