# Supplementary material for: Validation of a Novel Mobile Application for Assessing Pediatric Tracheostomy Emergency Simulations
Source: OTO Open. 2024 Jul 4;8(3):e145. doi: 10.1002/oto2.145 (PMC11222740; doi:10.1002/oto2.145)
Supplement: Supplementary file 1 — Supplementary information. [file OTO2-8-e145-s003.docx]

**Supplemental Table 1.** Overview of Application Training Videos.

|  |  | **Training Video** | | |
| --- | --- | --- | --- | --- |
|  |  | “Excellent” | “Good” | “Poor” |
| **Time of Step^a^ (Seconds)** | Step 1  “Assesses Breathing” | 2:53 | 0:35 | 0:38 |
|  | Step 2 “Calls for Help” | 0:20 | 1:24 | 4:45 |
|  | Step 3 “Suctions” | 0:44 | 1:00 | 1:00 |
|  | Step 4  “Trach Problem” | 1:09 | 1:16 | 1:40 |
|  | Step 5  “Trach Size-Length-Type” | 0:18 | 1:22 |  |
|  | Step 6  “Maskable”^b^ |  |  |  |
|  | Step 7  “Shoulder Roll” | 1:18 |  |  |
|  | Step 8  “Removes Trach” | 1:31 | 2:42 |  |
|  | Step 9  “Bag Mask” | 1:25 | 2:16 | 1:31 |
|  | Step 10  “Inserts Trach” | 1:54 | 3:32 |  |
|  | Step 11  “Trach Vent” | 2:01 | 3:42 |  |
|  | Step 12  “Trach Ties” | 2:38 | 4:36 |  |

a, occurrence and timing of steps determined through consensus video review by two authors as reference standard.

b. step 6 was identified for inclusion after production of training videos and thus was not modeled.

**Supplemental Table 2.** Initial Measures of Accuracy by Video and Step.

| **Variable** | **Time-based  Agreement, %** | **Step Occurrence Agreement, %** | **Average Difference from Reference Standard, Sec** ± **SD (Number of timestamp comparisons)** |
| --- | --- | --- | --- |
| Video^a^ |  |  |  |
| 1 | 88.9 | 93.1 | -1.9 ± 10.9 (41) |
| 2 | 80.6 | 94.4 | +7.4 ± 26.7 (34) |
| 3 | 83.3 | 93.1 | -6.8 ± 34.1 (37) |
| 4 | 90.3 | 95.8 | -3.6 ± 15.9 (21) |
| 5 | 83.3 | 94.4 | +2.8 ± 3.4 (39) |
| 6 | 83.3 | 87.5 | +0.9 ± 3.7 (28) |
| 7 | 81.9 | 84.7 | +1.7 ± 2.6 (18) |
| 8 | 94.4 | 100.0 | +7.0 ± 24.5 (42) |
| 9 | 81.9 | 91.7 | +1.4 ± 9.9 (55) |
| 10 | 91.7 | 93.1 | +2.9 ± 8.0 (15) |
| Step^b^ |  |  |  |
| 1 | 85.0 | 96.7 | -1.0 ± 9.0 (46) |
| 2 | 90.0 | 91.7 | +1.1 ± 1.9 (38) |
| 3 | 88.3 | 93.3 | +1.9 ± 18.4 (48) |
| 4 | 48.3 | 81.7 | +6.0 ± 41.7 (45) |
| 5 | 75.0 | 80.0 | +17.9 ± 32.1 (7) |
| 6 | 95. | 96.7 | +3.0 ± 3.2 (4) |
| 7 | 98.3 | 98.3 | +1.6 ± 1.5 (5) |
| 8 | 91.7 | 98.3 | -0.7 ± 4.8 (41) |
| 9 | 93.3 | 98.3 | -0.1 ± 4.1 (18) |
| 10 | 81.7 | 85.0 | -2.0 ± 12.8 (30) |
| 11 | 95.0 | 100.0 | ~~-~~0.8 ± 3.3 (36) |
| 12 | 90.0 | 93.3 | +3.4 ± 3.3 (12) |

a**,** all steps and raters; b, all videos and raters.

**Supplemental Table 3.** Generalized Linear Mixed-Effects Regression Results for Time-based Agreement, Step 4 Excluded.

| **Variable** | **Predicted Time-based Agreement, % (95% CI)** | **OR of Predicated Time-based Agreement (95% CI)** | **p-value** |
| --- | --- | --- | --- |
| Video^a^ |  |  | .19 |
| 1 | 94 (81 - 98) | 0.08 (0.005 - 1.11) | .06 |
| 2 | 93 (79 - 98) | 0.06 (0.004 - 0.85) | .04 |
| 3 | 95 (82 - 99) | 0.08 (0.005 - 1.22) | .07 |
| 4 | 99 (93 - 100) | 0.46 (0.02 - 9.25) | .61 |
| 5 | 92 (75 - 98) | 0.05 (0.003 - 0.73) | .03 |
| 6 | 94 (79 - 98) | 0.07 (0.005 - 1.02) | .05 |
| 7 | 92 (76 - 97) | 0.05 (0.003 - 0.72) | .03 |
| 8 | 100 (95 - 100) | *Ref* | *Ref* |
| 9 | 95 (83 - 99) | 0.08 (0.006 - 1.26) | .07 |
| 10 | 99 (94 - 100) | 0.68 (0.03 - 15.54) | .81 |
| Rater Experience^b^ |  |  | .008 |
| ENT | 98 (96 - 99) | *Ref* | *Ref* |
| CC | 94 (89 - 97) | 0.29 (0.13 - 0.63) | .002 |
| MS | 96 (92 - 98) | 0.46 (0.21 - 1.02) | .05 |
| Step^c^ |  |  | .17 |
| 1 | 93 (78 - 98) | 0.54 (0.08 - 3.61) | .52 |
| 2 | 96 (85 - 99) | 1.01 (0.14 - 7.39) | .99 |
| 3 | 96 (83 - 99) | 0.88 (0.12 - 6.43) | .90 |
| 5 | 83 (61 - 94) | 0.21 (0.03 - 1.27) | .09 |
| 6 | 99 (91 - 100) | 3.13 (0.31 - 32.04) | .34 |
| 7 | 100 (95 - 100) | 8.67 (0.53 - 142.75) | .13 |
| 8 | 96 (85 - 99) | *Ref* | *Ref* |
| 9 | 98 (89 - 100) | 1.70 (0.21 - 13.76) | .62 |
| 10 | 92 (75 - 98) | 0.48 (0.07 - 3.26) | .45 |
| 11 | 99 (91 - 100) | 2.73 (0.29 - 25.68) | .38 |
| 12 | 96 (85 - 99) | 1.10 (0.15 - 8.18) | .93 |

Abbreviation: ENT, pediatric otolaryngology attending physician; CC, pediatric critical care attending physician; MS, 3rd or 4th year medical student.

a, all raters and 11 steps (excluding 4); b, all videos and 11 steps (excluding 4); c, all videos and raters.

**Supplemental Figure 1A-C.** Number of Time-based Agreements with Reference Standard by Rater (All Videos and Steps). A: ENT; B: CC; C: MS.

Abbreviation: ENT, pediatric otolaryngology attending physician; CC, pediatric critical care attending physician; MS, 3rd or 4th year medical student.
