# Supplementary figures and images for: Validation of a Novel Mobile Application for Assessing Pediatric Tracheostomy Emergency Simulations
Source: OTO Open. 2024 Jul 4;8(3):e145. doi: 10.1002/oto2.145 (PMC11222740; doi:10.1002/oto2.145)

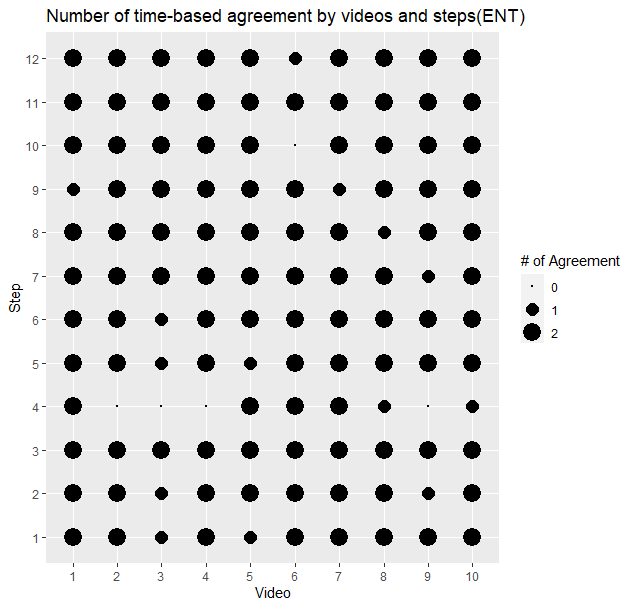

Supplement: Supplementary file 2 — Supplementary information. [file OTO2-8-e145-s002.tiff]

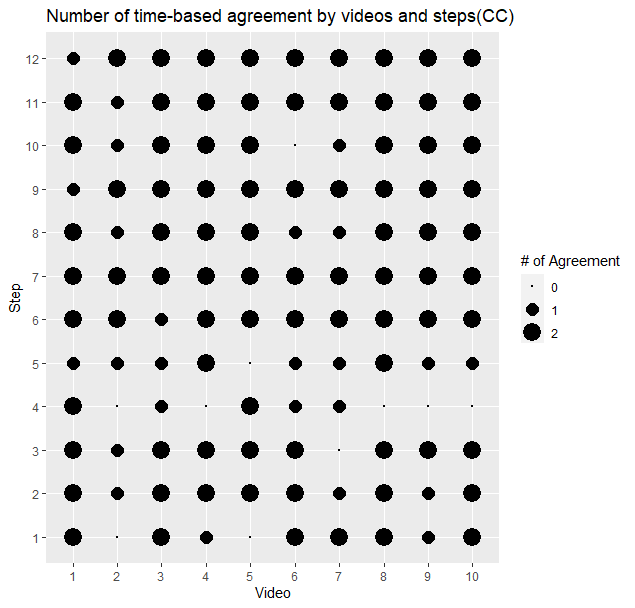

Supplement: Supplementary file 3 — Supplementary information. [file OTO2-8-e145-s001.tiff]

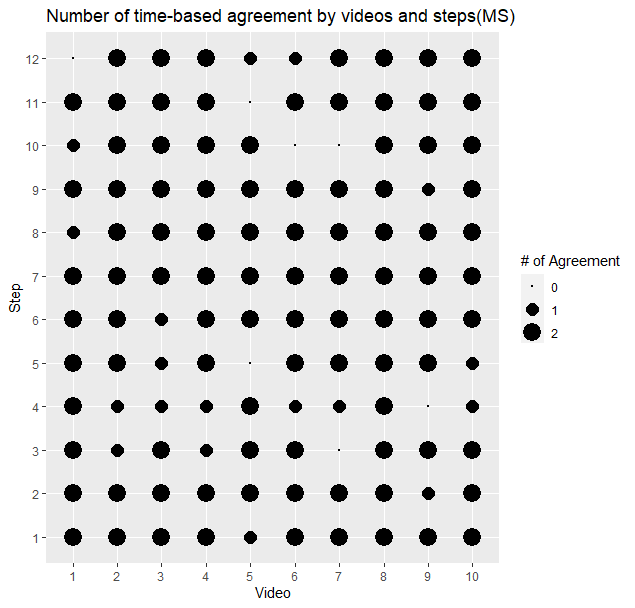

Supplement: Supplementary file 4 — Supplementary information. [file OTO2-8-e145-s004.tiff]
